# Singing from the Grave: DNA from a 180 Year Old Type Specimen Confirms the Identity of *Chrysoperla carnea* (Stephens)

**DOI:** 10.1371/journal.pone.0121127

**Published:** 2015-04-08

**Authors:** Ben W. Price, Charles S. Henry, Andie C. Hall, Atsushi Mochizuki, Peter Duelli, Stephen J. Brooks

**Affiliations:** 1 Life Sciences Department, Natural History Museum, London, England; 2 Department of Ecology and Evolutionary Biology, University of Connecticut, Storrs, Connecticut, United States of America; 3 Core Research Laboratories, Natural History Museum, London, England; 4 National Institute for Agro-Environmental Sciences, Tsukuba City, Ibaraki, Japan; 5 WSL Swiss Federal Research Institute, Birmensdorf, Switzerland; Biodiversity Insitute of Ontario—University of Guelph, CANADA

## Abstract

Historically serving as repositories for morphologically-based taxonomic research, natural history collections are now increasingly being targeted in studies utilizing DNA data. The development of advanced molecular techniques has facilitated extraction of useable DNA from old specimens, including type material. Sequencing diagnostic molecular markers from type material enables accurate species designation, especially where modern taxonomic hypotheses confirm morphologically cryptic species complexes. One such example is *Chrysoperla carnea* (Stephens), which belongs to a complex of about 20 cryptic species, most of which can only be reliably distinguished by their pre-mating courtship songs or by DNA analysis. The subtle morphological variation in the group has led to disagreement over the previous designation of the lectotype for *C*. *carnea*, an issue that has been further compounded because *Chrysoperla carnea* is a highly valued biological control agent in arable crops. Archival DNA extraction and sequencing from the 180 year old lectotype specimen, combined with Bayesian and Likelihood based phylogenetic analyses of modern specimens from the entire complex, were used to establish unambiguously the true identity of *Chrysoperla carnea*.

## Introduction

Natural history collections are a treasure trove of biological diversity, where millions of individual organisms, collected by generations of taxonomists, reside in perpetuity awaiting further examination as taxon concepts evolve. In recent times this research has increasingly included the use of molecular data, as improved methods of amplification have resulted in the successful retrieval of short DNA fragments from archival material [e.g., 1, 2–9] stored up to 194 years since collection [10]. Indeed, the success of archival DNA studies has resulted in the techniques being applied to type specimens, even where the type is the only known specimen [e.g. hummingbird: 11] or where the goal is to assign recently collected specimens, identified using molecular or acoustic methods, to the correct species [e.g. leafhoppers and butterflies: 12, 13].

One insect group in which traditional morphological approaches have been augmented by both acoustic and molecular data is the *Chrysoperla carnea*-group of green lacewings (Insecta: Neuroptera: Chrysopidae). This group comprises a complex of about 20 cryptic species distributed throughout the Northern Hemisphere and Afrotropics [14]. Some members of the species complex, particularly *Chrysoperla carnea* (Stephens, 1835), are widely used as biocontrol agents in arable and citrus crops. Until the early 1990s, *C*. *carnea* was thought to be a single morphologically variable species distributed across most of the Palaearctic region. Work by Henry and colleagues [15, 16] has now shown that this taxon includes a swarm of morphologically similar species that can only be reliably distinguished by analysis of their pre-mating courtship songs (duets).

**Fig 1:**
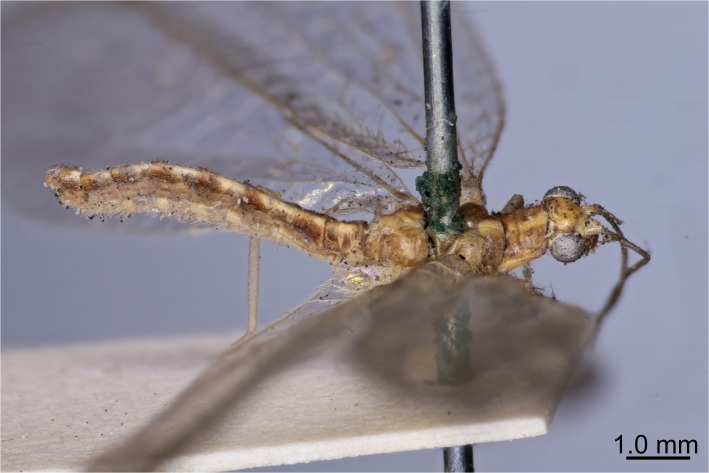
Lectotype of *Chrysoperla carnea* (Stephens). Mounted female specimen (voucher: BMNH(E) 1239048) is shown before dissection and abdomen extraction.

*Chrysoperla carnea* was originally described from a short type series collected by James Francis Stephens in London and Scotland in the early 19th century [17]. These specimens are now deposited in the Natural History Museum, London. A female lectotype for *C*. *carnea* was designated by Leraut [18] from this series (Fig. 1), as well as a lectotype for *C*. *affinis*, also described originally by Stephens [17]. Analysis of living British specimens in the *C*. *carnea*-group by Henry *et al*. [19, 20] revealed the presence of three species, all of which co-occur in the London area and can be distinguished on the basis of their unique courtship songs. One of these, *C*. *lucasina* (Lacroix, 1912), can be diagnosed morphologically by the presence of a prominent dark brown lateral stripe on the pleural membrane at the base of the abdomen. This marking is absent in the other two species. The remaining two species (*C*. *pallida* Henry *et al*., 2002 and *C*. *carnea*) can be reliably distinguished from each other by clear differences in their courtship songs [20] and mitochondrial DNA profiles [21]. The only morphological character that will consistently distinguish specimens of *C*. *carnea* from *C*. *pallida* is the shape of the genital lip on sternite 8+9 of males. Unfortunately, the lectotype of *C*. *carnea* is a female. Other morphological characters that are not sex-related, including tarsal claw morphology, colour and extent of head markings, and colouration of abdominal sternal setae, can be used to assign some specimens to the correct species, but there is considerable overlap and mixing of character states.

The aged and bleached female lectotype (Fig. 1) shares some morphological characteristics with *C*. *pallida* (i.e., pale abdominal setae and lightly marked maxillary stipes) and others with *C*. *carnea* (i.e., dimensions of the tarsal claw). The other specimens in the *C*. *carnea* type series, especially the males and including the female lectotype of *C*. *affinis* (Stephens, 1835), exhibit morphological characters that unambiguously identify them as *C*. *carnea* [20]. Stephens [17] coined the name ‘carnea’ to reflect the reddish-brown to purplish winter coloration of the adults he had collected. In contrast, *C*. *pallida* turns yellowish-brown during the winter [22]. For these reasons, Henry *et al*. [20] attributed the name *C*. *carnea* to the song-type informally known as Cc4 or ‘motorboat’ [23]. This nomadic species is widespread in crop environments and is widely used as a biocontrol agent, whereas *C*. *pallida* (formerly song-type Cc2 or ‘slow-motorboat’) prefers deciduous forests and rarely occurs in open agricultural situations. The decision by Henry *et al*. [20], therefore, was intended to maintain stability in the literature. Not all authors have accepted this decision. For more than twenty years there has been a taxonomic controversy whenever a paper on green lacewings in agriculture has been reviewed for publication. Canard and Thierry [24] and Thierry *et al*. [25, 26], for example, maintain that the lectotype of *C*. *carnea* is actually an example of song-type Cc2 and that, therefore, *C*. *pallida* is a synonym of *C*. *carnea*. As these authors assert, it would then follow that the name *C*. *affinis* should be assigned to specimens of song-type Cc4.

The mitochondrial DNA of *C*. *carnea* and *C*. *pallida* is distinct, however, so in an attempt to settle the controversy regarding the true identity of *C*. *carnea* we have extracted and analysed DNA from the 180 year old lectotype of *C*. *carnea*. The results show clearly that Henry *et al*. [20] were correct in assigning the name *C*. *carnea* to the taxon they designated as song type Cc4.

## Materials and Methods

### DNA extraction and sequencing

**Fig 2:**
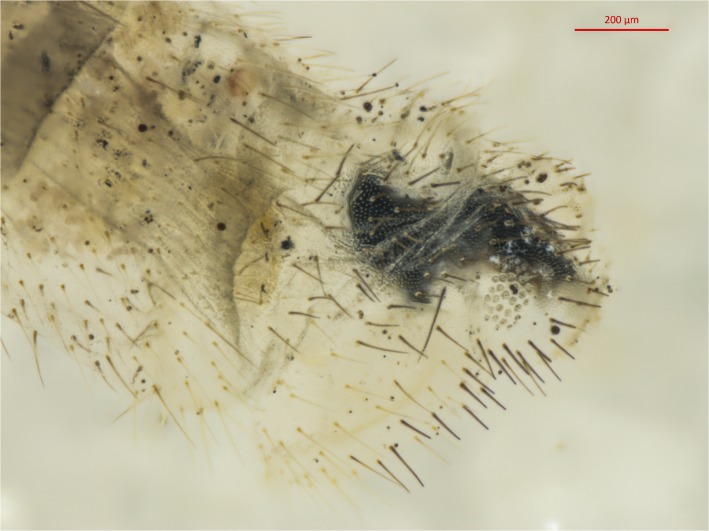
Terminalia of *Chrysoperla carnea* lectotype. Tip of abdomen is shown following DNA extraction.

Specimens were imaged before and after dissection (Figs. 1 and 2). A non-type specimen from the Stephen’s collection (BMNH(E) 1239047) was used to validate the method prior to attempting the extraction from the lectotype specimen (BMNH(E) 1239048). All lab equipment and areas were cleaned with DNA Away surface decontaminant (Thermo Scientific), and new consumables and reagents were used to prevent cross contamination of DNA between extraction of the test specimen and subsequent extraction of the lectotype. In each case a single leg was removed and genomic DNA was extracted using the Qiagen QIAamp DNA Micro kit as per manufacturer’s protocol “Isolation of genomic DNA from tissues”, modified by initially grinding the leg with a plastic micro-pestle in 20μl 1xTE buffer before the addition of lysis buffer (180 μl buffer ALT and 20μl proteinase K). The tissues were incubated at 56°C overnight (approx. 17 hours). In addition 1μg of carrier RNA was added to buffer AL at the appropriate stage. Cytochrome oxidase subunit (COI) sequences were amplified in eight separate reactions using the primer pairs designed by referring to COI sequences of the genus *Chrysoperla* deposited in the DDBJ/EMBL/GenBank database (S1 Table, S1 Fig.) in addition to C1-J-1718 and TL2-N-3014 [27]. Each reaction consisted of 1mM total dNTPs, 3mM MgCl_2_, 1.25u Bio-Taq DNA polymerase (Bioline), 0.1μM each primer and 1x reaction buffer (67mM Tris-HCl, 16mM (NH_4_)_2_SO_4_, 10mM KCl). Cycling conditions were: initial denaturation 94°C for 1 m followed by 40 cycles of 94°C for 30 s, 50°C for 30 s and 72°C for 30 s, with a final elongation of 10 m at 72°C. To increase the potential DNA yield from the lectotype the abdomen was extracted separately from the leg using the Qiagen QIAamp DNA Micro kit as per manufacturer’s protocol with the following modifications to minimise damage to external morphology: the abdomen was removed and soaked whole in buffer ATL with proteinase K at 56°C overnight, rather than ground or vortexed. 1μg of carrier RNA was added to buffer AL. Following extraction the abdomen was washed in 500μl of TE buffer, then in a series of TE/ethanol dilutions (50% ethanol for 5 hours at 4°c then 70% ethanol for 16 hours at 4°c) before being stored in 100% ethanol and imaged with a Zeiss Axio Zoom v.16 stereo microscope (Fig. 2). All PCR products were cleaned using Millipore PCR filter purification plates as per manufacturer’s instructions, then sequenced bi-directionally using BigDye terminator reaction mix v3.1 in a 3730xl DNA analyser (Applied Biosystems) at the NHM sequencing facility.

### Sequence analysis

Fragments of COI were individually analysed in comparison to the BOLD COI “Full Database” (boldsystems.org). As a result of non-barcoding regions of COI being successfully retrieved from the specimens the NCBI database (http://blast.ncbi.nlm.nih.gov/Blast.cgi) was queried using Mega BLAST, optimised to search for highly similar sequences. Sequence fragments were combined to create contigs for both the test and lectotype specimen and aligned with data corresponding to positions 238–1464 of the COI gene data from song-verified identifications of the 15 published and 5 unpublished but distinct species in the *Chrysoperla carnea*-group and three outgroup species selected from the *Chrysoperla pudica*-group [16, 21, 28]. Sequences were then compared manually and by using pairwise estimates of the proportion of differences (p-distance) in MEGA v.6 [29], with standard errors estimated using 1000 bootstrap replicates.

### Phylogenetic analysis

For phylogenetic analysis, the COI data from the test and lectotype specimens were added to the most comprehensive molecular dataset of the genus *Chrysoperla* available: the four-gene mitochondrial dataset of Henry *et al*. that includes ND2, COI, COII, and ND5 ([14, 16, 21] for 23 species (DDBJ accession numbers AB671778–AB672105, AB836669–AB836680, and AB981362–AB981369). Two alignments were used in the analyses: the COI-only dataset and the combined four-gene mitochondrial dataset. Data were partitioned using the greedy algorithm in PartitionFinder v1.1.1 [30] and analysed under a Bayesian framework using MrBayes 3.2.2 [31] following the methods outlined previously [16]. In addition, the data were analysed under a likelihood framework using eight separate best tree searches and a separate 100 replicate bootstrap analysis in GARLI 2.0 [32]. All analyses were run on the Cipres Science Gateway [33]. The COI and four-gene mitochondrial data (analysis and resulting tree files) are available on the NHM data portal: http://dx.doi.org/10.5519/0059186.

## Results

The leg of the test specimen provided 552 base pairs (bp) of mitochondrial DNA sequence in two fragments (Fragment 1: 278 bp, COI position 238–516, GenBank Accession Number: KP117071; Fragment 7: 274 bp, COI position 998–1271, GenBank Accession Number: KP117072). None of the eight fragments designed for the study were amplified from the lectotype leg; however, the novel combination of primers C-CI-505F and C-CI-578R yielded a 125 bp fragment with one base assignable to *Chrysoperla carnea* (sequence not published). The whole-abdomen extraction provided 466 base pairs in two fragments (Fragment 4: 226 bp, COI position 717–942, GenBank Accession Number: KP117073; Fragment 8: 240 bp, position 1225–1464, Genbank Accession Number: KP117074).

### BOLD/Blast searches

Only Fragment 1 amplified from the test specimen included any overlap (278 bp) with the Folmer “barcode region” of COI. Analysis of this fragment within the BOLD database resulted in *C*. *carnea* as the only match with 100% sequence identity (30 sequences), followed by representatives of *C*. ‘adamsi-K’ (99.64% match); *C*. *lucasina* (98.91% match); and *C*. *agilis* Henry et al., 2003, *C*. *mediterranea* (Hölzel, 1972), *C*. *pallida* and *C*. *nipponensis* (Okamoto, 1914) (all 98.55%). Separate BLAST searches of each of the four fragments resulted in a 100% match only with *C*. *carnea* in all cases (S2 Table).

### Sequence distances

Sequences were compared between the test and lectotype specimens and the three candidate species that occur in the UK, whose identifications were verified by song analysis: *C*. *carnea*, *C*. *pallida* and *C*. *lucasina* (S3 Table). Of the 20 parsimony informative sites in the COI alignment, ten distinguish *C*. *carnea* from *C*. *pallida*, six distinguish *C*. *carnea* from *C*. *lucasina* and three distinguish *C*. *pallida* from *C*. *lucasina* (S3 Table).

In addition, the proportion of differences (p-distance) between the lectotype sequence and each of the 20 species in the *carnea*-group was calculated in MEGA v.6 using pairwise comparisons and standard errors estimated with 1000 bootstrap replicates (S2 Fig.). The lectotype specimen is more similar to *C*. *carnea* (~0.1% difference) than to any other species in the group, almost by an order of magnitude (~0.1% lectotype:*C*. *carnea* vs ~0.9–2.6% lectotype:remaining *carnea*-group), including the other two candidate UK species *C*. *lucasina* (~1.2% difference) and *C*. *pallida* (~1.9% difference).

### Phylogenetic analyses

The greedy algorithm in PartitionFinder selected five partitions as the optimum partitioning strategy for phylogenetic analysis (S4 Table), and the most appropriate available models were used in all Bayesian and Likelihood analyses. When analysing the COI gene alone the data were partitioned into the three codon positions and modelled under the models selected by PartitionFinder for each codon (S4 Table).

Both Likelihood and Bayesian analyses of the combined (4630 bp) mitochondrial data returned a tree with a very similar topology to that of Henry *et al*. [21], showing a well-supported North American clade sister to *C*. *nipponensis* and in general a poorly resolved Eurasian polytomy (S3 Fig.). Of the Eurasian species only *C*. *carnea*, *C*. “adamsi K”, *C*. *renoni* (Lacroix, 1933) and *C*. *pallida* comprised well supported monophyletic clades. Both the test and lectotype specimens fell within the *C*. *carnea* clade with 100% bootstrap and 0.93 posterior probability support.

**Fig 3:**
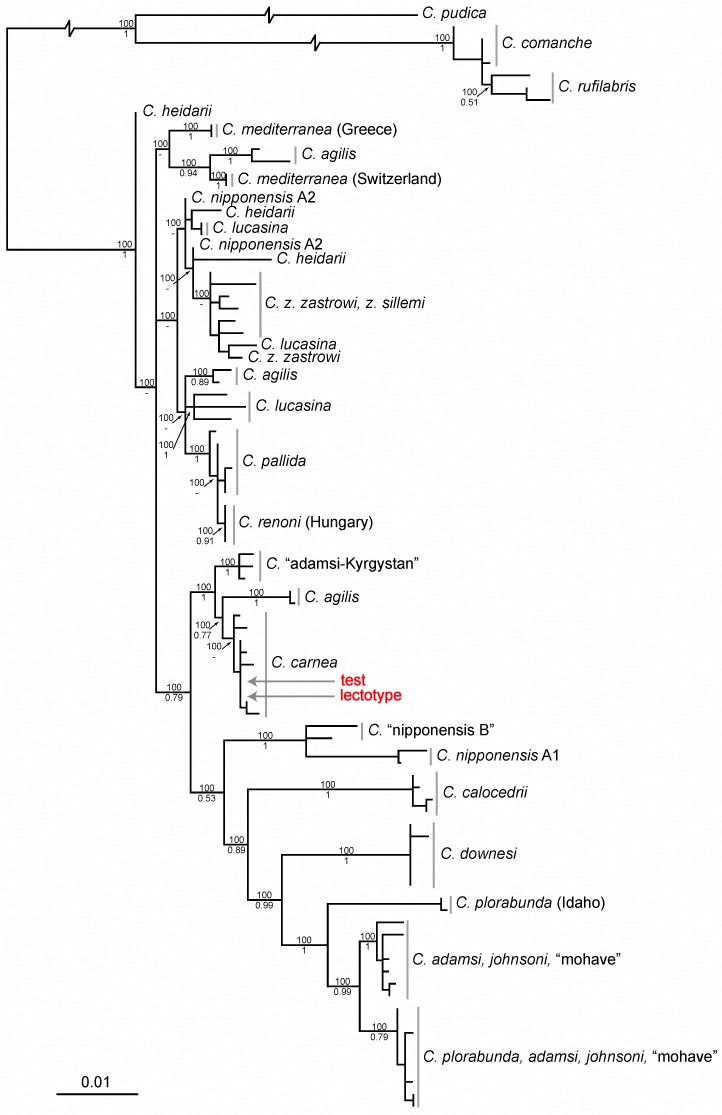
Maximum Likelihood phylogram of the cryptic species of the *Chrysoperla carnea*-group. Phylogram is based on analysis of 1226 bp of COI sequence. Numbers at the branch points are bootstrap support (above) and Bayesian posterior probabilities (below); branch lengths are proportional to the number of substitutions per site except where indicated. Positions of the test and lectotype specimens in the phylogram are shown in red.

Likelihood and Bayesian analysis of the COI data alone (Fig. 3) did not significantly affect the topology in comparison to the combined mitochondrial dataset; however, support values were reduced across the tree. Both the test and lectotype specimens fell within the *C*. *carnea* clade with 100% bootstrap and 0.77 posterior probability support. Data files (analysis and resulting tree files) available on the NHM data portal: http://dx.doi.org/10.5519/0059186.

## Discussion

While much of the previous work has shown the utility of both ancient [7] and archival [1–10] insect specimens for DNA analysis, the number of studies on insect type material is far fewer [12, 13]. The present study adds to the literature on the use of DNA to analyse archival type material in solving current taxonomic issues, and has shown that standard molecular methods can also be applied to type material of soft-bodied insects, such as lacewings, which are small and extremely fragile. Dried, mounted specimens can yield viable DNA even 180 years after collection from as little as a single leg, as shown in the test specimen. Although the DNA from these specimens was highly fragmented, requiring multiple independent PCR amplifications, the rapid advance of molecular techniques will enable easier extraction of DNA data from old museum specimens. In addition, the falling cost of next generation sequencing will facilitate retrieval of larger portions of the genome from these old specimens in future [9].

Direct DNA comparison of the lectotype specimen with published sequences of all species within the *C*. *carnea*-group (S2 Table, S3 Table, S2 Fig. and S3 Fig.) leaves no doubt that the lectotype of *C*. *carnea* Stephens is the same taxon as ‘Cc4/motorboat’ [20]. Of the three candidate species present in Britain, DNA from the lectotype is almost identical to ‘Cc4/motorboat’ [i.e. *C*. *carnea* sensu 20] and easily separable from *C*. *pallida* and *C*. *lucasina* based on the number of base changes (S3 Table) and its well-supported placement on the phylogram (Fig. 3). While *C*. *carnea* and *C*. *pallida* may be difficult to distinguish morphologically, they are easily differentiated using both DNA and acoustic characters. The multi-gene mitochondrial analysis of the *carnea*-group (S3 Fig.) highlights the high taxonomic divergence between *C*. *carnea* and *C*. *pallida* despite their similar morphology, further underpinning the need for molecular and acoustic studies to understand evolutionary relationships in the group.

The results of DNA analysis of the lectotype of *C*. *carnea* clearly and unambiguously demonstrate that Henry *et al*. [20] were correct in assigning specimens of the taxon determined acoustically as ‘Cc4/motorboat’ to *C*. *carnea* and that *C*. *pallida* is not a synonym of *C*. *carnea*, as asserted by Thierry *et al*. [25, 26]. Our results, once and for all, settle the debate over the true identity of *C*. *carnea*, the most important lacewing species in arable crops in the Western Palaearctic. The arboreal species considered to be *C*. *carnea* by Canard and Thierry [24] and Thierry *et al*. [25] is confirmed as *C*. *pallida* [20].

Analysis of COI data (Fig. 3) and multi-gene mitochondrial data (S3 Fig.), confirm that the *C*. *carnea*-group of lacewings has a complicated evolutionary history, with multiple species sharing mitochondrial haplotypes resulting in polyphyletic species entities. Our results confirm that additional analyses of nuclear DNA will be required to gain a robust understanding of the evolutionary history of these species. Furthermore our results indicate that traditional “DNA Barcoding” (i.e. based on the 5’ end of COI data alone) is not feasible for species identification in this group in general. For example, of the 20 cryptic species recognized to date [14], 15 cannot be positively identified from barcode data because of paraphyly or polyphly (Fig. 3) resulting from incomplete lineage sorting or mitochondrial capture [34]. It is important to note however that COI data can be used to distinguish between the three British species, particularly *C*. *carnea* and *C*. *pallida*, which both form distinct, well-supported monophyletic clades (Fig. 3). This result agrees with recent work on the Neuroptera fauna of Bavaria which has confirmed that COI barcode data can distinguish *C*. *carnea* from *C*. *pallida* and *C*. *lucasina*, but that the latter two may be indistinguishable using barcode data alone [35].

## Conclusion

DNA data from the lectotype confirm unambiguously the true identity of *C*. *carnea*, stabilising the taxonomy and nomenclature of this taxon. *Chrysoperla pallida* is a valid distinct species and can be distinguished from *C*. *carnea* using morphological [20], acoustic [20] and molecular (Fig. 8 in [21]) methods. The name *C*. *affinis* is a junior synonym of *C*. *carnea* [28]. This confirms that Henry *et al*. [20] were correct in assigning the name *C*. *carnea* to the taxon they designated as song type Cc4. This study also confirms that *C*. *carnea* is the correct name for the species most widely used in biocontrol in arable crop environments in the western Palaearctic. Finally this study has successfully recovered DNA from one of the oldest and most fragile pinned insect museum specimens to date. Whilst this 180 year old lacewing can no longer perform its mating song, its DNA has empowered it to ‘sing from the grave’ and solve a long-standing taxonomic conundrum.

## Supporting Information

S1 Fig Primer position and orientation on the COI reference sequence. Novel primers are designated “F” (forward) and “R” (reverse). The position of the Folmer “barcoding” primers LCO 1490 and HCO 2198 are shown for comparison.
(TIF)

S2 Fig Mean percentage sequence difference (%) between the lectotype specimen of *C*. *carnea* and other lacewing taxa. The other taxa included all 15 published species and 5 distinct but not yet formally described species of the *Chrysoperla carnea*-group, as well as the three outgroup species. Error bars indicate standard errors estimated with 1000 bootstrap replicates.
(TIF)

S3 Fig Maximum Likelihood phylogram of the cryptic species of the *Chrysoperla carnea*-group, based on 4630 bp of mtDNA sequence data. Numbers at the branch points are bootstrap support (above) and Bayesian posterior probabilities (below); branch lengths are proportional to the number of substitutions per site except where indicated. Positions of the test and lectotype specimens in the phylogram are shown in red.
(TIF)

S1 Table Primers and combinations used to amplify the eight fragments of COI. (DOCX)

S2 Table Comparison of BLAST search results between fragments and the three candidate species present in the UK. (DOCX)

S3 Table Summary of informative sites in the 1226 bp of COI sequenced from the three candidate species present in the UK. Characters which can be used to diagnose the species in pairwise comparison (marked ✓), variable characters (marked red X), characters present in the sequenced fragments of the lectotype and test specimen (marked *). Character position corresponds to the position on the COI reference sequence.
(DOCX)

S4 Table Partitions and corresponding models as identified by PartitionFinder. Where this model was unavailable the next most appropriate model was used for analyses.
(DOCX)
