# Supplementary figures and images for: Singing from the Grave: DNA from a 180 Year Old Type Specimen Confirms the Identity of Chrysoperla carnea (Stephens)
Source: PLoS One. 2015 Apr 8;10(4):e0121127. doi: 10.1371/journal.pone.0121127 (PMC4390323; doi:10.1371/journal.pone.0121127)

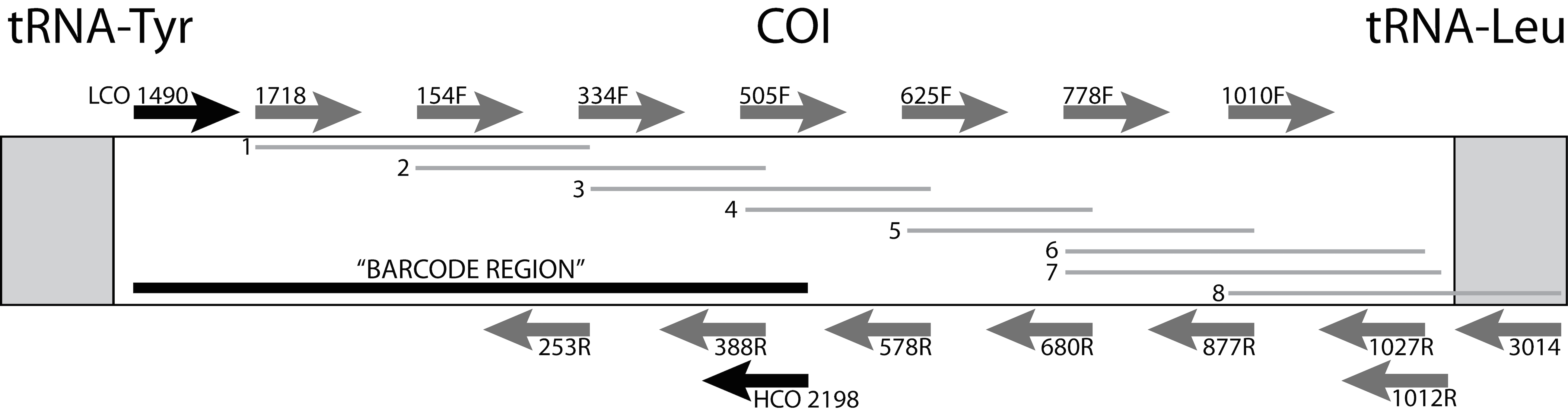

Supplement: S1 Fig — Novel primers are designated “F” (forward) and “R” (reverse). The position of the Folmer “barcoding” primers LCO 1490 and HCO 2198 are shown for comparison. (TIF) [file pone.0121127.s001.tif]

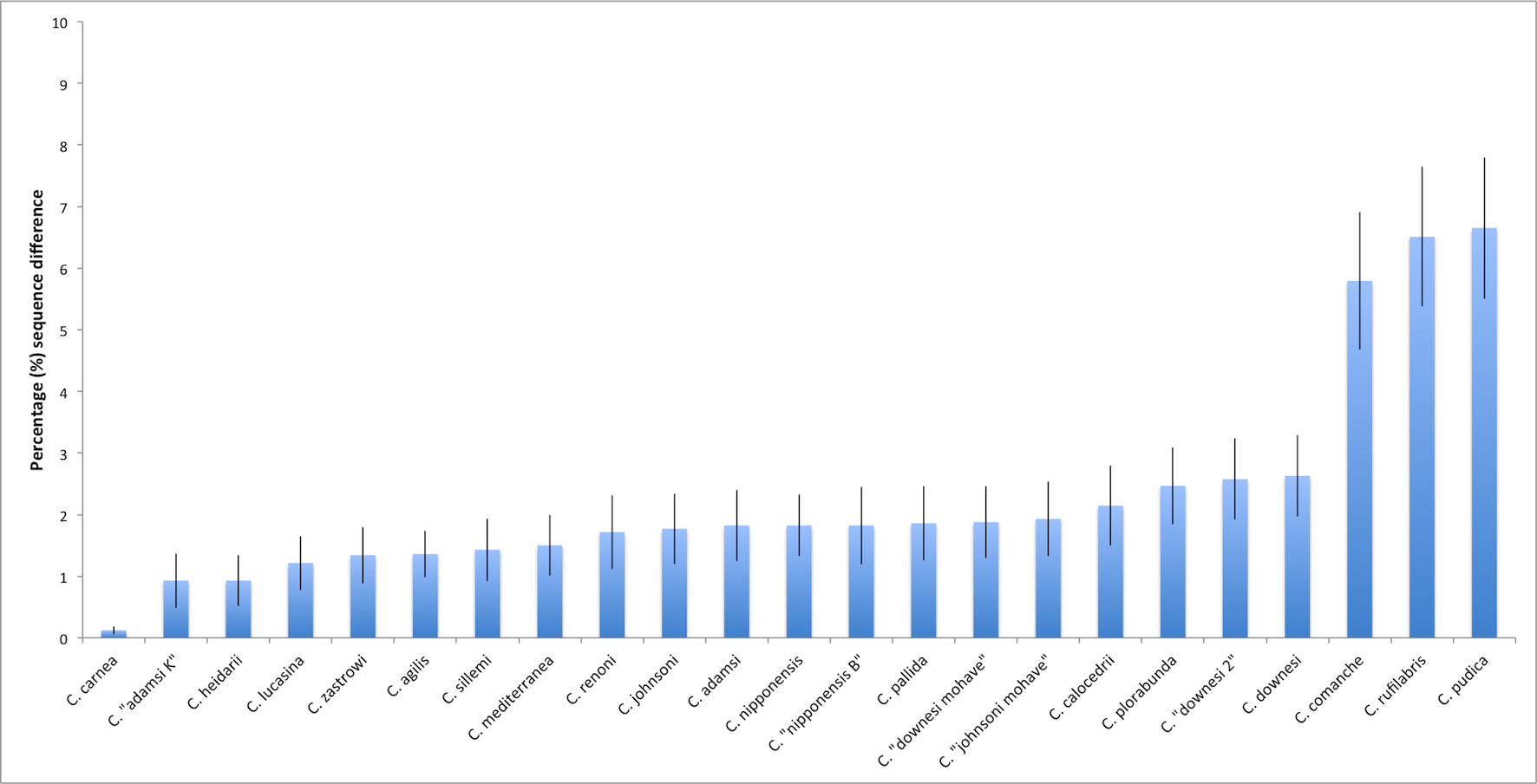

Supplement: S2 Fig — The other taxa included all 15 published species and 5 distinct but not yet formally described species of the Chrysoperla carnea-group, as well as the three outgroup species. Error bars indicate standard errors estimated with 1000 bootstrap replicates. (TIF) [file pone.0121127.s002.tif]

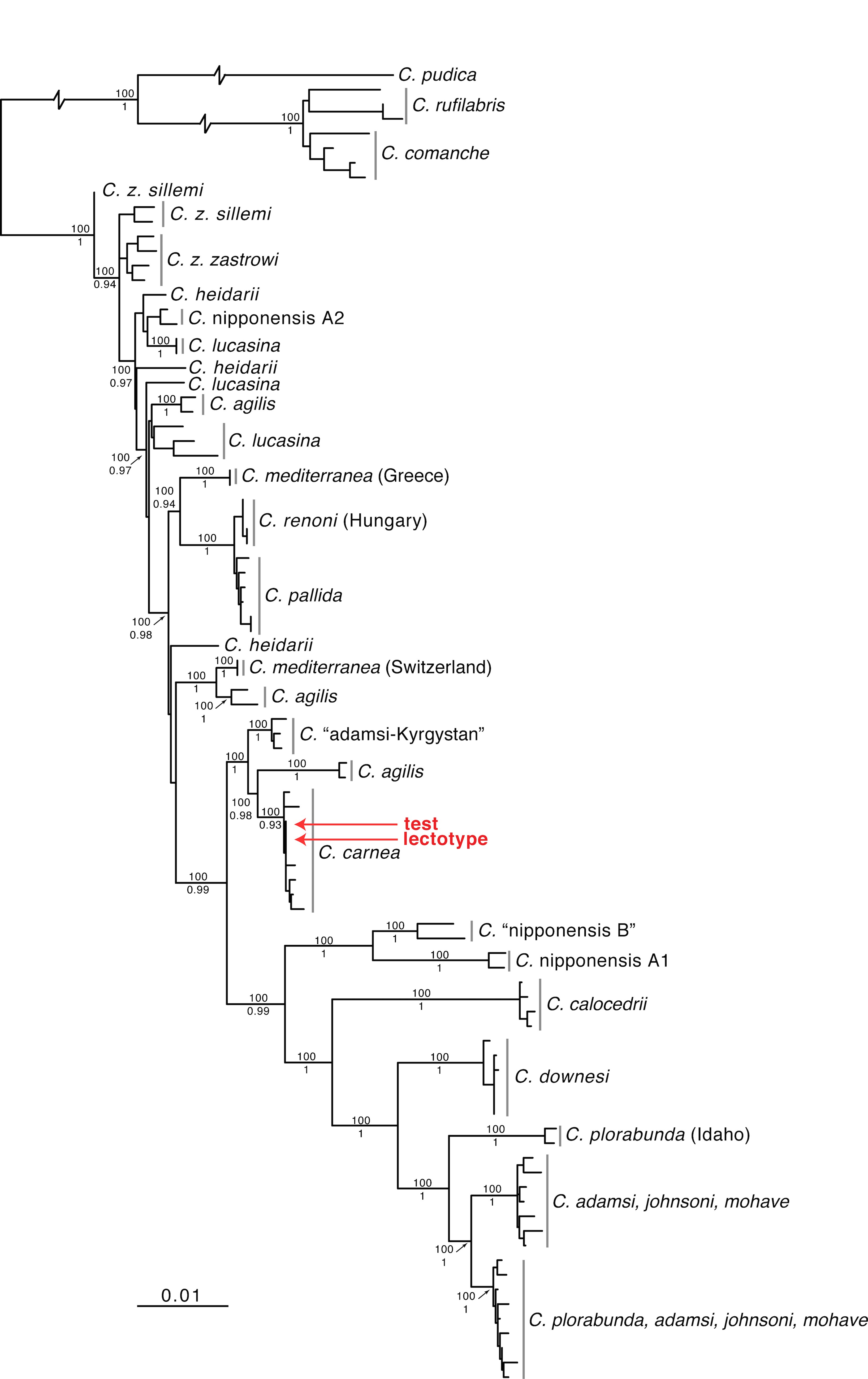

Supplement: S3 Fig — Numbers at the branch points are bootstrap support (above) and Bayesian posterior probabilities (below); branch lengths are proportional to the number of substitutions per site except where indicated. Positions of the test and lectotype specimens in the phylogram are shown in red. (TIF) [file pone.0121127.s003.tif]
