# Supplementary material for: Singing from the Grave: DNA from a 180 Year Old Type Specimen Confirms the Identity of Chrysoperla carnea (Stephens)
Source: PLoS One. 2015 Apr 8;10(4):e0121127. doi: 10.1371/journal.pone.0121127 (PMC4390323; doi:10.1371/journal.pone.0121127)
Supplement: S1 Table — (DOCX) [file pone.0121127.s004.docx]

**S1 Table: Primers and combinations used to amplify the eight fragments of COI.**

| **Fragment** | **Forward primer** | **Sequence** | **Reverse primer** | **Sequence** |
| --- | --- | --- | --- | --- |
| 1 | C1-J-1718 | GGAGGATTTGGAAATTGATTAGTTCC | C-CI-253R | ACTGATCATACAAATAATGGTATAC |
| 2 | C-CI-154F | ATGCTGGAGCTTCTGTTGATTTAGCTA | C-CI-388R | TGTTGATATAAAATTGGGTCTCCTCC |
| 3 | C-CI-334F | CATTACCTGTATTAGCTGGTGCTATTA | C-CI-578R | CCAACAGTAAATATATGATGAGCTC |
| 4 | C-CI-505F | GTCATATTATTGCTCAAGAAAGTGG | C-CI-680R | GTTCCATGTAAAGTAGCTAATCAAC |
| 5 | C-CI-625F | TTGGAATAGATGTTGATACTCGAGC | C-CI-877R | CAATGAACAAATCCTGCTATAATAGC |
| 6 | C-CI-778F | ATTTACTGTTGGAGGATTAACTGG | C-CI-1012R | GAATAACGACGRGGTATTCCAGCTAA |
| 7 | C-CI-778F | ATTTACTGTTGGAGGATTAACTGG | C-CI-1027R | CAGGATAATCAGAATAACGACG |
| 8 | C-CI-1010F | CATTTTTCCCTCAACATTTTTTAGG | TL2-N-3014R | TCCAATGCACTAATCTGCCATATTA |
