# Supplementary material for: Singing from the Grave: DNA from a 180 Year Old Type Specimen Confirms the Identity of Chrysoperla carnea (Stephens)
Source: PLoS One. 2015 Apr 8;10(4):e0121127. doi: 10.1371/journal.pone.0121127 (PMC4390323; doi:10.1371/journal.pone.0121127)
Supplement: S2 Table — (DOCX) [file pone.0121127.s005.docx]

**S2 Table:** **Comparison of BLAST search results between fragments and the three candidate species present in the UK.**

|  | ***C. carnea*** | | | ***C. pallida*** | | | ***C. lucasina*** | | |
| --- | --- | --- | --- | --- | --- | --- | --- | --- | --- |
| **Fragment** | **Max Score** | **E-Value** | **% match** | **Max Score** | **E-Value** | **% match** | **Max Score** | **E-Value** | **% match** |
| 1 (test) | 514 | 3E-140 | 100% | 492 | 1E-135 | 99% | 497 | 2E-137 | 99% |
| 7 (test) | 507 | 2E-142 | 100% | 490 | 3E-135 | 99% | 479 | 7E-132 | 98% |
| 4 (type) | 418 | 2E-113 | 100% | 401 | 2E-108 | 99% | 407 | 4E-110 | 99% |
| 8 (type) | 444 | 3E-121 | 100% | 416 | 6E-113 | 98% | 427 | 3E-116 | 99% |
