# Supplementary material for: Singing from the Grave: DNA from a 180 Year Old Type Specimen Confirms the Identity of Chrysoperla carnea (Stephens)
Source: PLoS One. 2015 Apr 8;10(4):e0121127. doi: 10.1371/journal.pone.0121127 (PMC4390323; doi:10.1371/journal.pone.0121127)
Supplement: S3 Table — Characters which can be used to diagnose the species in pairwise comparison (marked ✓), variable characters (marked red X), characters present in the sequenced fragments of the lectotype and test specimen (marked *). Character position corresponds to the position on the COI reference sequence. (DOCX) [file pone.0121127.s006.docx]

**S3 Table: Summary of informative sites in the 1226 bp of COI sequenced from the three candidate species present in the UK.** (see Supporting Information Captions)

| Character  position | Possible  States | *C. carnea /*  *C. pallida* | *C. carnea /*  *C. lucasina* | *C. lucasina /*  *C. pallida* | Lectotype | Test |
| --- | --- | --- | --- | --- | --- | --- |
| 271 | CT | ✓ | ✓ | ✗ |  | * |
| 364 | AT | ✓ | ✓ | ✗ |  | * |
| 406 | CT | ✓ | ✓ | ✗ |  | * |
| 433 | AG | ✓ | ✗ | ✗ |  | * |
| 622 | AG | ✗ | ✗ | ✗ |  |  |
| 775 | CT | ✓ | ✗ | ✓ | * |  |
| 892 | AG | ✓ | ✗ | ✗ | * |  |
| 913 | AC | ✓ | ✓ | ✗ | * |  |
| 1018 | AG | ✗ | ✗ | ✗ |  | * |
| 1084 | CT | ✗ | ✗ | ✗ |  | * |
| 1099 | AG | ✗ | ✗ | ✗ |  | * |
| 1105 | AG | ✗ | ✗ | ✗ |  | * |
| 1111 | AG | ✗ | ✗ | ✗ |  | * |
| 1147 | CT | ✗ | ✗ | ✗ |  | * |
| 1249 | AGT | ✓ | ✓ | ✗ | * | * |
| 1264 | CT | ✓ | ✓ | ✗ | * | * |
| 1282 | CT | ✗ | ✗ | ✓ | * |  |
| 1324 | AG | ✗ | ✗ | ✗ | * |  |
| 1366 | CT | ✗ | ✗ | ✗ | * |  |
| 1438 | AG | ✓ | ✗ | ✓ | * |  |
