# Supplementary material for: Singing from the Grave: DNA from a 180 Year Old Type Specimen Confirms the Identity of Chrysoperla carnea (Stephens)
Source: PLoS One. 2015 Apr 8;10(4):e0121127. doi: 10.1371/journal.pone.0121127 (PMC4390323; doi:10.1371/journal.pone.0121127)
Supplement: S4 Table — Where this model was unavailable the next most appropriate model was used for analyses. (DOCX) [file pone.0121127.s007.docx]

**S4 Table: Partitions and corresponding models as identified by PartitionFinder.** (see Supporting Information Captions)

.

| Partition | Model  Selected | Model  Applied | Codon Positions |
| --- | --- | --- | --- |
| 1 | TrN+G | GTR+G | COII posn. 3; ND2 posn. 3 |
| 2 | TrN+I+G | GTR+I+G | ND2 posn. 1; ND5 posn. 1 |
| 3 | HKY+I+G | HKY+I+G | COII posn. 2; COI posn. 2; ND2 posn. 2; ND5 posn. 2 |
| 4 | TrN+I+G | GTR+I+G | COII posn. 1, COI posn. 1 |
| 5 | K81uf+G | GTR+G | COI posn. 3, ND5 posn. 3 |
